# Driving Force Analysis of Natural Wetland in Northeast Plain Based on SSA-XGBoost Model

**DOI:** 10.3390/s23177513

**Published:** 2023-08-29

**Authors:** Hanlin Liu, Nan Lin, Honghong Zhang, Yongji Liu, Chenzhao Bai, Duo Sun, Jiali Feng

**Affiliations:** 1 College of Marine Engineering, Dalian Maritime University, Dalian 116026, China; liuhanlin@dlmu.edu.cn (H.L.); baichenz@163.com (C.B.); 1120220125sd@dlmu.edu.cn (D.S.); 0203212x@dlmu.edu.cn (J.F.); 2 School of Geomatics and Prospecting Engineering, Jilin Jianzhu University, Changchun 130018, China; linnan@jlju.edu.cn (N.L.); liuyongji@jlju.edu.cn (Y.L.); 3 School of Earth Science, Jilin University, Changchun 130026, China; 4 Geological Survey Institute of Jilin Province, Changchun 130102, China

**Keywords:** Northeast China Plain, spatiotemporal evolution, driving force, SSA-XGBoost

## Abstract

Globally, natural wetlands have suffered severe ecological degradation (vegetation, soil, and biotic community) due to multiple factors. Understanding the spatiotemporal dynamics and driving forces of natural wetlands is the key to natural wetlands’ protection and regional restoration. In this study, we first investigated the spatiotemporal evolutionary trends and shifting characteristics of natural wetlands in the Northeast Plain of China from 1990 to 2020. A dataset of driving-force evaluation indicators was constructed with nine indirect (elevation, temperature, road network, etc.) and four direct influencing factors (dryland, paddy field, woodland, grassland). Finally, we built the driving force analysis model of natural wetlands changes to quantitatively refine the contribution of different driving factors for natural wetlands’ dynamic change by introducing the sparrow search algorithm (SSA) and extreme gradient boosting algorithm (XGBoost). The results showed that the total area of natural wetlands in the Northeast Plain of China increased by 32% from 1990 to 2020, mainly showing a first decline and then an increasing trend. Combined with the results of transfer intensity, we found that the substantial turn-out phenomenon of natural wetlands occurred in 2000–2005 and was mainly concentrated in the central and eastern parts of the Northeast Plain, while the substantial turn-in phenomenon of 2005–2010 was mainly located in the northeast of the study area. Compared with a traditional regression model, the SSA-XGBoost model not only weakened the multicollinearity of each driver but also significantly improved the generalization ability and interpretability of the model. The coefficient of determination (*R*^2^) of the SSA-XGBoost model exceeded 0.6 in both the natural wetland decline and rise cycles, which could effectively quantify the contribution of each driving factor. From the results of the model calculations, agricultural activities consisting of dryland and paddy fields during the entire cycle of natural wetland change were the main driving factors, with relative contributions of 18.59% and 15.40%, respectively. Both meteorological (temperature, precipitation) and topographic factors (elevation, slope) had a driving role in the spatiotemporal variation of natural wetlands. The gross domestic product (GDP) had the lowest contribution to natural wetlands’ variation. This study provides a new method of quantitative analysis based on machine learning theory for determining the causes of natural wetland changes; it can be applied to large spatial scale areas, which is essential for a rapid monitoring of natural wetlands’ resources and an accurate decision-making on the ecological environment’s security.

## 1. Introduction

Wetlands are ecosystems with the highest natural biodiversity and one of the indispensable living spaces for human beings [1,2,3]. Wetlands can regulate and store floods, purify water, degrade pollution, and beautify the environment. They also play an important role in maintaining ecological balance and biodiversity [4,5,6,7,8,9,10,11]. Since the 20th century, global climate change and human activities have made the restoration and management of wetlands more complex. Different stress sources in the development of various types of wetlands have diversified the evolution trends of wetland ecosystems. These changes have profoundly impacted the balanced development of regional ecosystems [12,13,14,15]. The system combining remote sensing (RS) and geographic information systems (GIS) has become an important information extraction and variation monitoring method for wetlands of different scales, because of the powerful spatial analysis capacity in large-scale wetland monitoring and driving factor analysis [16,17,18,19,20,21]. A series of studies in the Harike region of Punjab, India, and the Negemet region of western Ethiopia support the effectiveness of joint RS and GIS [22,23].

Exploratory research on wetland spatiotemporal evolution patterns has made continuous progress due to the rapid development of RS and GIS, and research on the causes of natural wetlands variation is gradually becoming a hot issue in the field of natural wetlands [24,25,26]. Natural wetlands are particularly sensitive to human activities (urbanization, long-term groundwater extraction, species invasion, and industrial pollution) [27,28] and are influenced by natural factors such as climate change and topographic conditions [29,30,31,32]. Due to the lack of effective methods to control excessive human activity, the fifth assessment reports from the IPCC (Intergovernmental Panel on Climate Change) predicted further global warming trends, including the temperature rising, a precipitation regime shift, a sea level rise, and an increasing frequency of extreme climate events [33,34,35]. Previous studies have confirmed that the main driving factors for wetlands include precipitation, temperature, elevation, slope, population, and GDP (gross domestic product), which significantly influence the spatial and temporal variations of wetlands [36,37,38]. Therefore, constructing a perfect indicator system of driving factors is the primary issue in wetland variation analysis. Regarding driving factors’ selection, most scholars often select influencing factors with a high similarity across different study areas [38,39]. Utilizing driving factors selected from previous research may reflect some correlation between these factors and a variation in natural wetlands. However, some indicators do not consider the indirect effects, such as potential agricultural and industrial development behind the population and GDP growth [40]. Achieving this joint characterization across both temporal and spatial dimensions can thus prove difficult, such as the temporal invariance of the elevation and the regional statistical features of the population. The complement of road networks and typical land types (dryland, paddy field, woodland, grassland) can effectively compensate for the shortcomings of traditional driving factors [41]. They can be used not only to analyze past utilization patterns of natural wetlands but also to capture information on spatial and temporal changes of natural wetlands, which is very suitable for the quantitative evaluation of the contribution of multiple factors to wetland change [42]. Notably, the drivers of wetland change often exhibit significant spatial and temporal heterogeneity. Driving force analysis models based on mathematical and statistical theories can better understand the underlying causes of wetland change. Statistical methods commonly used for wetland change analysis include the partial correlation analysis [43,44], geographic detectors [45,46,47], geographically weighted regression [48,49,50], and gray correlation [51,52,53]. While the above methods can provide insight into the degree of association between each driver and wetland variation, they are limited in mitigating multicollinearity between variables within high-dimensional datasets. As a result, the calculated values can be heavily influenced by bias, leading to potentially misleading results.

In contrast to the weak interpretability of traditional linear regression models, which indirectly calculate relative contribution rates using regression coefficients, ensemble learning models based on decision trees have significant advantages [54]. They can sort the contributions of raw input feature attributes and visually display the influential optimal factors, which are widely used in forest pest control, land use classification, and air pollutant detection [55]. Random forest and gradient-boosting decision trees are ensemble learning algorithms commonly used to handle high-dimensional data, nonlinear relationships, and dynamic weights. However, as the size of the input data increases, these models still carry certain disadvantages, such as a slow training speed, poor generalization ability, and difficulty handling nonstandard, missing, and outlier values [56]. To address the inefficiency problem in complex engineering, Chen et al. proposed the extreme gradient boosting algorithm (XGBoost) in 2016. This method is a scalable and efficient form of gradient boosting algorithm, which mainly uses a more regularized model architecture than gradient boosting to prevent model training overfitting [57]. In addition, the XGBoost algorithm decides how to handle missing values by counting the nodal distribution pattern of all missing values and still effectively supports the data imbalance in the sampling process of each influencing factor [58].

The Northeast China Plain is the largest plain and an important commercial grain production base in China. Since the 1950s, climate change and human activities have led to intensified droughts and floods, a drastic variation in natural wetlands resource, destabilized ecosystems, and an increasingly apparent trend of ecosystem function degradation [59]. Given the importance and necessity of natural wetlands’ protection and restoration, it is necessary to link natural wetlands’ transformations to different influencing factors. Based on six periods of remote sensing data of natural wetlands from 1990 to 2020, this study defines the spatial and temporal variation characteristics and internal transformation mechanisms of natural wetlands in the Northeast Plain of China through the perspectives of the dynamic index, transfer matrix, and transfer intensity. Additionally, we construct a dataset of driving force evaluation indicators based on nine indirect and four direct factors. Meantime, the mathematical statistical model is used to quantitatively calculate the contribution of different factors to natural wetlands’ evolution. In particular, the objectives were (1) to illustrate the spatiotemporal and transfer characteristics of natural wetlands in the Northeast China Plain during 1990–2020; (2) to select road networks and four typical land types, combined with spatially balanced sampling methods to improve the driving force evaluation index system from the time scale and spatial scale; and (3) to construct a natural wetland variation driving force analysis model based on the SSA parameter intelligent optimization algorithm and the XGBoost machine learning algorithm which verifies the feasibility of applying machine learning theory to natural wetland driver research.

## 2. Materials and Methods

### 2.1. Study Area

**Figure 1:**
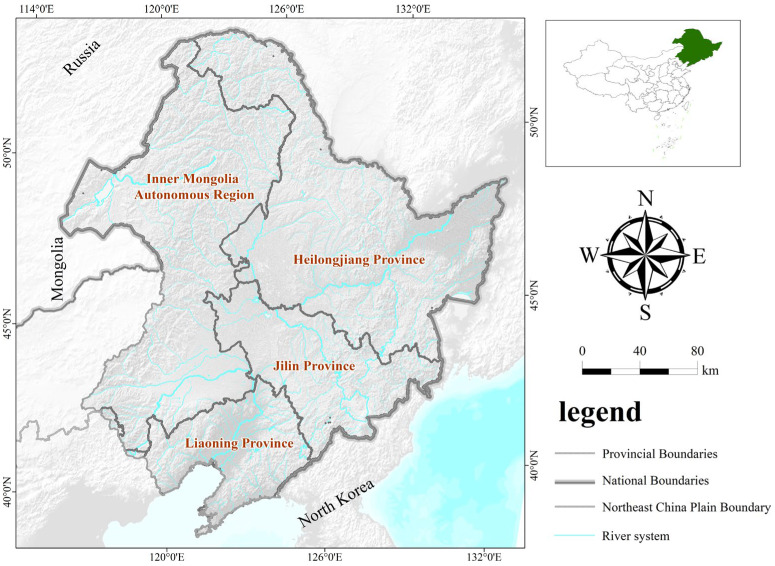
Location of the Northeast China Plain.

As one of the three major plains in China, the Northeast China Plain spans the four provinces of Heilongjiang, Liaoning, Jilin, and Inner Mongolia. It lies from 115°30′ E to 135°20′ E and 38°43′ N to 53°30′ N, covering a total area of about 1.24 million square kilometers. The study area is surrounded by mountains in the north, east, and west and the Liaodong Bay in the south. The altitude of the study area gradually decreases from north to south, averaging at 200 m. The topography of the study area includes piedmont diluvial/alluvial plains and platforms. The study area has a temperate continental monsoon climate, making it the transitional zone between China’s humid eastern monsoon region and the arid inland. The study area has four seasons: windy and dry spring and autumn, a summer with abundant rain, and a dry and cold winter. The average annual precipitation of the study area is greater than the evaporation. The multiyear average temperature ranges from −3.9 to 11.5 °C, the multiyear average precipitation ranges from 0.54 to 2.85 mm, and the accumulated temperature above 10 °C ranges from 1500 to 3700 °C. With a frost-free period of 90 to 160 d, the study area is suitable for growing crops such as corn, soybeans, spring wheat, and rice. The Northeast China Plain has widely distributed freshwater marshes and other types of wetlands such as river and lake wetlands. Among them, the river wetlands, mainly the Heilongjiang and Mudanjiang rivers, spread across the entire Northeast China Plain by forming a network of river systems. Lake wetlands such as Jingpo Lake and Wudalianchi Lake are concentrated in low-lying regions with a semihumid climate. Marsh wetlands are closely related to river wetlands and lake wetlands and are mainly distributed on riverbanks, around lakes, and between mountain valleys in the Daxinganling, Xiaoxinganling, and Changbai mountains (Figure 1).

### 2.2. Data Preparation

#### 2.2.1. Land Use and Land Cover Change (LUCC) Remote Sensing Dataset

**Figure 2:**
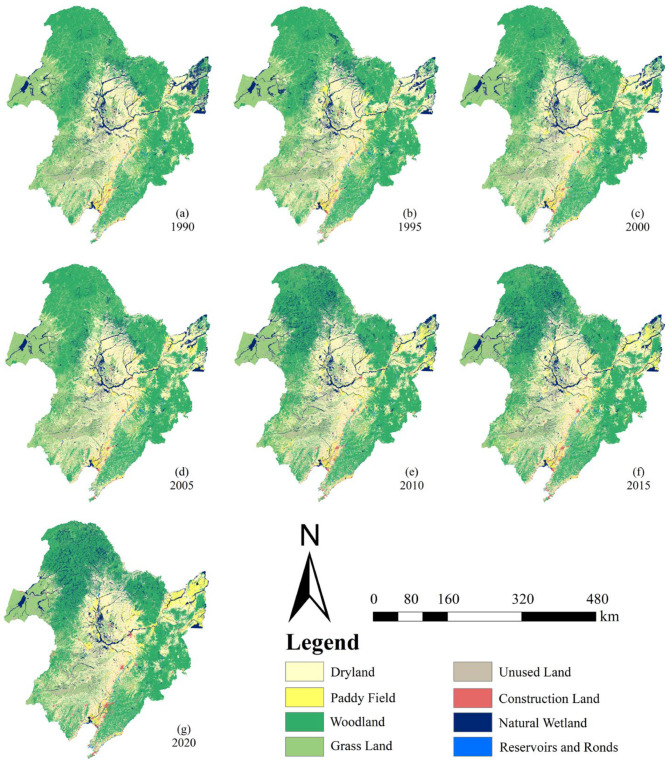
LUCC Data of Northeast Plain from 1990 to 2020.

LUCC data were obtained from the “China Multi-period Land Use Remote Sensing Monitoring Datasets” of the Resource and Environment Data Cloud Platform of the Chinese Academy of Sciences (http://www.resdc.cn/DOI) (accessed on 1 July 2021). The current dataset includes 1980, 1990, 1995, 2000, 2005, 2010, 2015, 2018, and the data production was generated by a manual visual interpretation using Landsat TM/ETM remote sensing images of each period as the main data source. Land-use types include 6 primary categories and 25 secondary categories, of which the primary categories consist of arable land, woodland, grassland, water surface, construction land, and unused land. Considering the periodicity of long-term time series data may affect the accuracy of research results, we supplemented the 2020 LUCC data for the Northeast Plains region with Landsat OLI remote sensing imagery and manual visual interpretation methods based on the 2018 data. In addition, to quantify more accurately the contribution of different land types to the spatiotemporal variation mechanism of natural wetlands, we reorganized the original 25 secondary land types into 8 primary categories according to the international “Wetland Convention” and the needs of this study. The results are shown in Figure 2.

**Figure 3:**
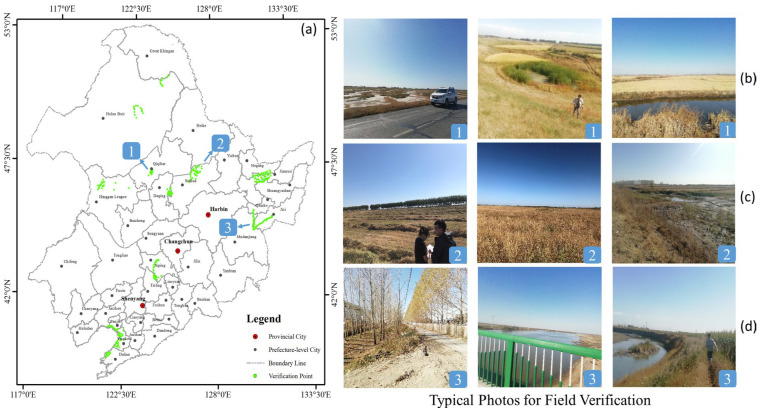
Field validation of LUCC data in 2020: (**a**) spatial distribution of checkpoints; (**b**) survey of agricultural activities in key reclamation areas; (**c**) survey of natural wetlands’ priority degraded areas; (**d**) survey of the road network and surface water.

Reliable data sources are essential for scientific research. To verify the accuracy of the added 2020 LUCC data, a total of 574 points to be verified were selected in the eastern, southern, and central regions as the primary verification areas in this study. The classification effect was tested by field verification. The results showed that the overall identification accuracy of each category in the region was over 85%, and the accuracy of grassland, woodland, and natural wetland was maintained at around 88.67%, proving that the data met the basic requirements for conducting the study (Figure 3).

#### 2.2.2. Driving Factor of Natural Wetland Variation

In this thesis, meteorology and topography were selected as the natural factors affecting wetland variation. Specifically, the topographical factors included elevation (ELE) and slope (SLO). Meteorological factors included temperature (TEM) and precipitation (PRE). Human activity factors included distance to the road network (DRN), population density distribution (PDD), gross domestic product (GDP), dryland (DRA), paddy field (PAF), woodland (WOL), and grassland (GRL) areas. Among them, four land categories are shown in Figure 2 and are not repeated here. In the description below, we use the abbreviated form of a driver as a proxy.

**Figure 4:**
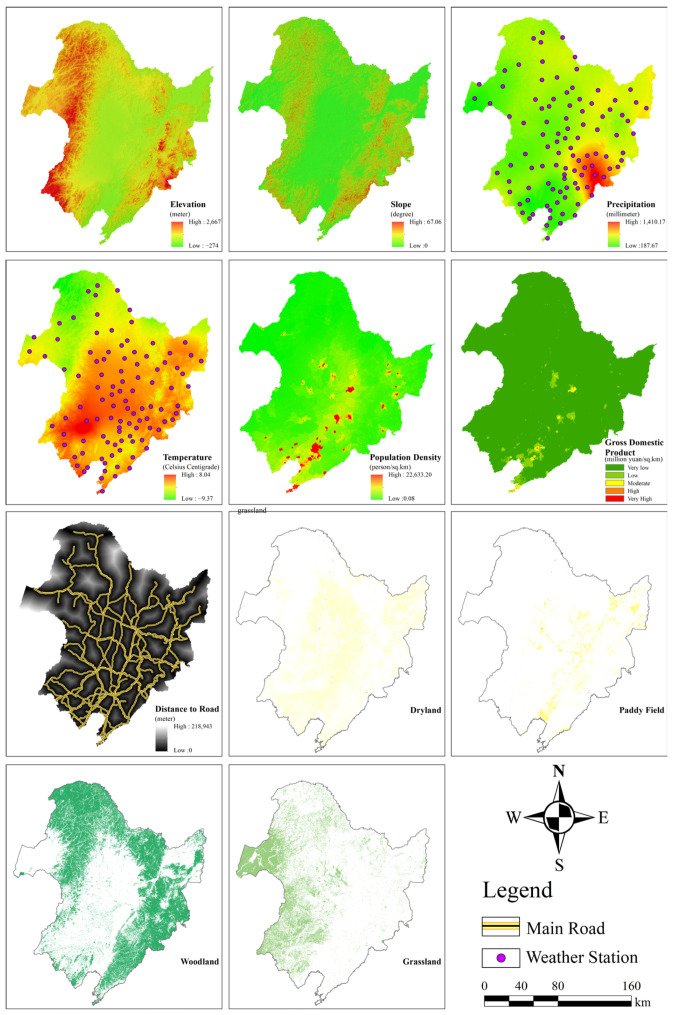
Spatial distribution of 11 factors driving natural wetland variation.

The ELE and SLO data for ASTER GDEM were from the USGS website (http://glovis/usgs.gov/) (accessed on 14 May 2021) with a spatial resolution of 30 m. Meteorological data were from the monthly dataset of China’s surface climate data of China National Meteorological Information Center and included the TEM (°C) and PRE (mm) at 113 meteorological stations inside or within the 50 km buffer zone of the Northeast China Plain from 1990 to 2019. The PDD, DRN, and GDP data were from the Resource and Environment Science and Data Center of the Chinese Academy of Sciences (https://www.resdc.cn/) (accessed on 14 May 2021). In this study, the spatial data of each driving factor needed to be preprocessed through techniques such as projection transformation, spatial interpolation, and raster clipping. Meanwhile each driving factor was resampled to the corresponding resolution to construct a natural-wetland multisource driving factor spatial dataset (Figure 4).

### 2.3. Spatiotemporal Variation of Natural Wetlands

#### 2.3.1. Dynamic Index of Natural Wetlands

The spatiotemporal variation characteristics of natural wetlands can be analyzed through the dynamic index. As an important indicator often used to measure wetland variation, the dynamic index quantitatively compares the differences in temporal and spatial variation between regions [60,61,62]. The method of expressing the variation degree of a certain wetland type is called single dynamic index, and the calculation formula is as follows:

(1) $$K=(U_{a}-U_{b})/U_{a}\times 1/T\times 100\%$$

where K is the dynamic index of wetland variation during the study period; $U_{a}$ and $U_{b}$ are the areas of a certain wetland type at the beginning of the study and the end of the study, respectively; and T represents the study period.

#### 2.3.2. Transfer Matrix of Natural Wetlands

Wetland variation is not only manifested as area changes but also the transformation among wetland types. Based on the RS interpretation data in 2000 and 2019, the interaction and evolution processes of various wetland types were discussed. Specifically, the transition matrix was selected to analyze the transformation direction, and the transformation intensity was selected to analyze the spatial distribution of transformation [63]. The transition matrix is as follows:

(2) $$S_{ij}=\left[\begin{array}{ccc} S_{11} & \cdots & S_{1n}\\ \vdots & \ddots & \vdots \\ S_{n1} & \cdots & S_{nn} \end{array}\right]$$

where $S_{ij}$ is the area transition matrix, S represents the area, n represents the wetland type, and *i* and *j* represent the wetland types in different periods, respectively.

The transformation intensity is a basic method to reflect the changes in spatial distribution [64,65]. It visualizes spatial differences by introducing the grid theory. Its mathematical expression can be defined as:

(3) $$R=\frac{\Delta S}{S_{grid}\times T}\times 100\%$$

where ΔS represents the area variation of each wetland type; $S_{grid}$ is the area of the established grid; T is the length of the study; R represents the transformation intensity within the unit grid.

### 2.4. Natural Wetland Driver Analysis Using SSA-Optimized XGBoost Model

#### 2.4.1. Sparrow Search Algorithm (SSA)

The sparrow search algorithm (SSA) optimizes model parameters by simulating the feeding and predator avoidance behavior of sparrows [66]. It has a strong optimization ability and fast convergence speed [45]. SSA can effectively reduce the search space, improve search efficiency, avoid local optimal solutions in the search space, and has a better optimization ability than other optimization algorithms such as PSO and GWO [67]. According to the study of Dong et al., the updated formula of the sparrow finder position is as follows [68]:

(4) $$X_{i,d}^{t+1}=\left\{\begin{array}{l} X_{i,d}^{t}\cdot \exp(\frac{-i}{\alpha \cdot G_{\max}}),R_{2}<ST\\ X_{i,d}^{t}+Q\cdot L,R_{2}\geq ST \end{array}\right.$$

where X is the sparrow position; *t* is the current update; *i* is the number of sparrows; *d* is the dimension; $G_{\max}$ is the maximum number of iterations; α is a random number following the uniform (0,1) distribution; *Q* is a random number that follows the standard normal distribution; *L* is a matrix where each element is 1; *ST* is the warning threshold with its value range of [0.5, 1]; *R*_2_ is the warning value.

The follower position update formula during the local search is [69]:

(5) $$X_{i,d}^{t+1}=\left\{\begin{array}{l} Q\cdot \exp(\frac{X_{w,d}^{t}-X_{i,d}^{t}}{i^{2}}),i>\frac{N}{2}\\ X_{b,d}^{t+1}+\left|X_{i,d}^{t}-X_{b,d}^{t+1}\right|\cdot A^{+}\cdot L,i\leq \frac{N}{2} \end{array}\right.$$

where $X_{w,d}^{t}$ is the worst position of the current population; $X_{b,d}^{t+1}$ is the optimal location of the current population; *A* is the 1×D matrix (row vector), and each element in the row vector is randomly assigned 1 or −1. 

The strategy for updating the location of scouts is as follows:

(6) $$X_{i,d}^{t+1}=\left\{\begin{array}{l} X_{b,d}^{t}+\beta \cdot \left|X_{i,d}^{t}-X_{best}^{t}\right|,f_{i}>f_{g}\\ X_{i,d}^{t}+\kappa \cdot (\frac{\left|X_{i,d}^{t}-X_{worst}^{t}\right|}{(f_{i}-f_{w})+\varepsilon }),f_{i}=f_{g} \end{array}\right.$$

where $X_{b,d}^{t}$ is the current global optimal position; β is the step control parameter; *k* is a uniform random number between [−1, 1]; $f_{i}$, $f_{g}$, and $f_{n}$ represent the fitness, global optimal fitness, and global worst fitness values of the current population, respectively; and ε is the smallest constant to avoid a zero division error.

#### 2.4.2. Extreme Gradient Boosting (XGBoost)

The traditional GBDT (gradient boosted tree) algorithm uses the idea of gradient descent and the criterion of minimizing the objective function to optimize the structure of the data computations. In 2015, Dr. Tianqi Chen improved on GBDT by constructing decision trees in a combination of parallel and serial trees, with a 2nd order expansion of the loss function based on adding *L*_1_ and *L*_2_ regularization equations. The XGBoost model is presorted before training [70]. It is called cyclically by saving it as a block structure, which significantly improves the computing efficiency and can be better applied to regression and classification problems [71]. In general, the regularized objective loss function f(L) for the *L*th iteration is shown in Equation (7):

(7) $$f(L)=\sum_{i=1}^{n}t(y^{\left(i\right)},{\overset{\wedge }{y_{L}}}^{\left(i\right)})+\sum_{j=1}^{L}\Omega (f_{j})$$

where *n* is the number of samples; ${\overset{\wedge }{y_{L}}}^{\left(i\right)}$ is the predicted value of the *i*th sample in *L* iterations; l is the original loss function; and Ω is the regularization function, which can be calculated from Equation (5):

(8) $$\Omega (f)=\gamma N+\frac{1}{2}\lambda \sum_{j=1}^{N}w_{j}^{2}$$

where *N* is the number of leaf nodes, and γ and λ denote the coefficients used to adjust the degree of regularization.

To better optimize the key parameters inside the regression model constructed by Equations (4) and (5), Equation (4) can be used to obtain the optimal solution of the objective function using a second-order Taylor expansion to improve the prediction accuracy of the *i*th iteration, and Equation (6) is shown below:

(9) $$f(L)=\sum_{i=1}^{n}\left[l(y^{\left(i\right)},{\overset{\wedge }{y_{L-1}}}^{\left(i\right)})+g_{i}\cdot f_{L}(x^{\left(i\right)})+\frac{1}{2}h_{i}\cdot f_{L}(x^{\left(i\right)})\right]+\Omega (f_{L})+K$$

where $g_{i}$ is $\partial_{{\hat{y}}_{L-1}}l(y^{\left(i\right)},{\hat{y}}_{L-1})$, $h_{i}$ is $\partial_{{}_{{\hat{y}}_{L-1}}}^{2}l(y^{\left(i\right)},{\hat{y}}_{L-1})$, representing the first-order derivative and second-order derivative of the loss function, respectively; *K* is the constant.

In terms of feature selection, the XGBoost model evaluates the rankings based on “gain” coverage and frequency, and uses the weights of the variables to rank the importance of the feature $v(X_{v})$, as shown in Equation (10):

(10) $$N_{v}=\sum_{r=1}^{L}\sum_{c=1}^{X=1}I(V_{L}^{c},v)$$

where *L* is the number of trees or iterations; $N_{v}$ is the number of leaf nodes of the *L*th tree; $V_{L}^{c}$ is a feature based on node *c*; *I*() is a schematic function; and ($V_{L}^{c},v$) can be calculated by Equation (11):

(11) $$(V_{L}^{c},v)=f(x)=\left\{\begin{array}{c} 1,if\;V_{L}^{c}=v\\ 0,\;otherwise \end{array}\right.$$

#### 2.4.3. *K*-Fold Cross-Validation (KCV)

**Figure 5:**
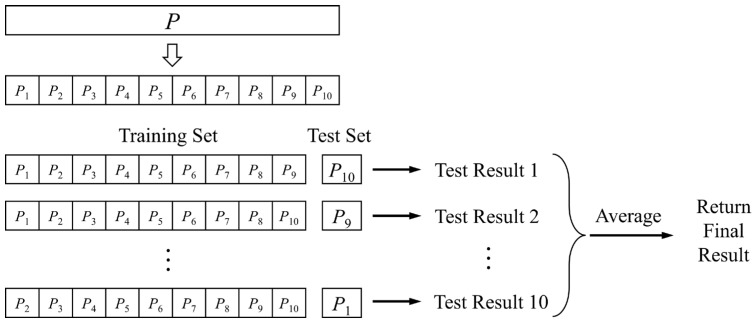
Tenfold cross-validation principle.

A statistical analysis technique called cross-validation is used to verify a classifier’s performance. The underlying idea is to divide the original dataset in two groups, with one serving as the validation set and the other as the training set. The classifier is first trained using the training set, and then the model that results from this training is tested using the validation set. The performance of the classifier is evaluated using this approach as a metric. In a *K*-fold cross-validation (KCV), the original data are divided into *K* groups, one of which is chosen to serve as the validation set for each iteration while the other *K* − 1 subsets are pooled to serve as the training set [72]. The structure is shown in Figure 5 for the case where *k* = 10.

## 3. Results

### 3.1. Natural Wetland Dynamics

From Table 1, it can be seen that the spatiotemporal variation of different land-use types in the Northeast Plain are relatively drastic, forming a multilevel transformation of land use structure primarily composed of dryland (DRA), woodland (WOL), grassland (GRL), and natural wetland (NAW), supplemented by paddy field (PAF), construction land (COL), unused land (UNL), and reservoirs and ponds (REP). The area of DRA shows a fluctuating upward trend, corresponding to the continuous decline of the GRL area, while the WOL area remains at around 500 × 10^3^ km^2^. The area of PAF, COL, and REP shows varying degrees of growth. Unlike other land types, the area of NAW and UNL has remained close over the past 30 years, showing a trend of first decreasing, then increasing, and an overall growth. In 2020, the total area of NAW was 104.79 × 10^3^ km^2^, accounting for 8.0% of the total study area, an increase of 32% compared with 79.27 × 10^3^ km^2^ in 1990. At the same time, during the research period from 2005 to 2010, NAW experienced a sudden change, with a leap amplitude of nearly 50%, which was much larger than that of other land types during the same period.

**Figure 6:**
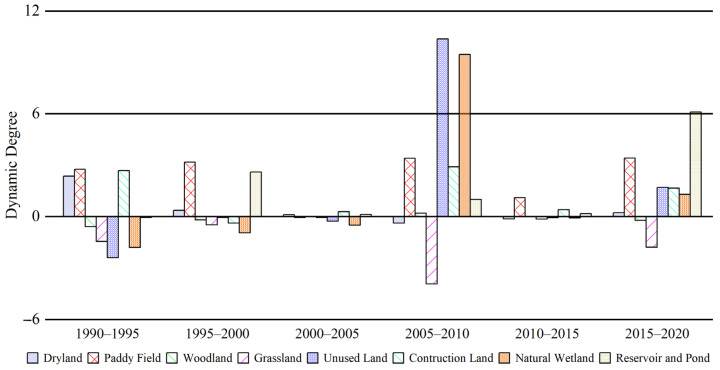
Dynamic degree of land use from 1990 to 2020.

From the comparison and analysis of the dynamics of different land-use types (Figure 6), the most drastic dynamic changes were observed during 2005–2010, with the rate of change significantly higher than that of other periods. This was mainly reflected in the sharp increase of UNL, which had a dynamic degree value exceeding 10.37% and a higher rate of change than other land types. Moreover, NAW also had a significantly higher dynamic degree value of 9.47% than other land types, showing the frequent phenomenon of conversion among different land types during that period. The dynamic degree of the remaining land types, including WOL, GRL, and DRA, remained below 5% and showed a relatively stable overall rate of change.

### 3.2. Natural Wetland Transfer Trends

**Figure 7:**
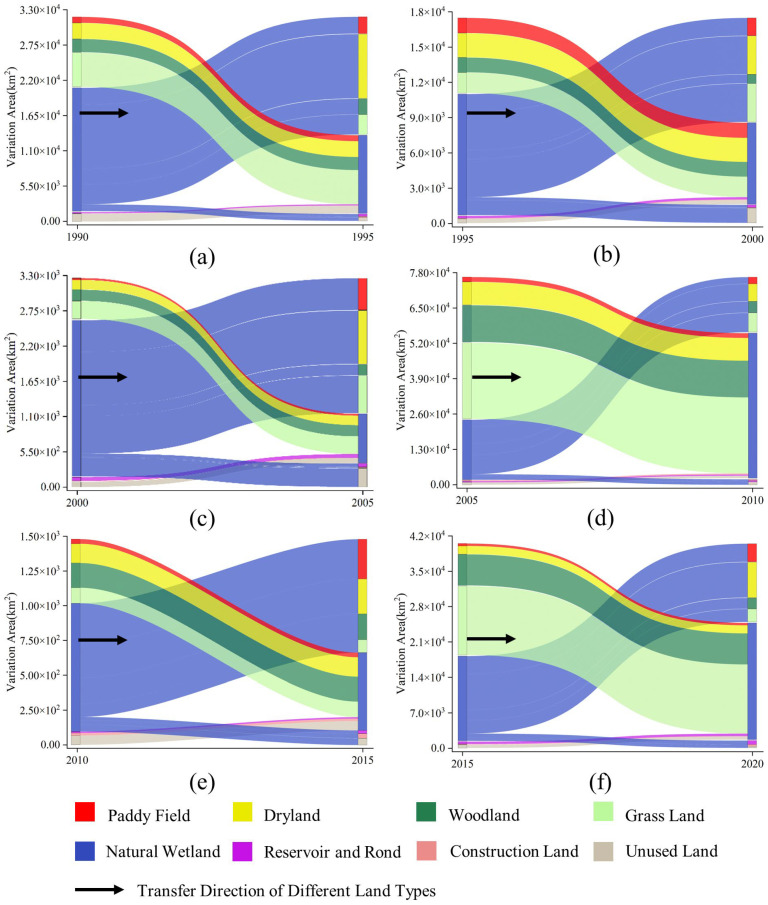
Transfer matrix of different land classes: (**a**) 1990–1995; (**b**) 1995–2000; (**c**) 2000–2005; (**d**) 2005–2010; (**e**) 2010–2015; (**f**) 2015–2020.

Based on the results of the above study, we obtained the spatiotemporal variation patterns of different land types in the Northeast Plain during the study cycle. However, since it was difficult to directly reflect the spatiotemporal transition trends within land-use types by a simple area increase or decrease, this paper used the transfer matrix to analyze the transition and variation intensity among land types from the time scale. The transition matrix was constructed based on a quantitative description of the system states and state transitions in the system analysis. The obtained results are depicted in Figure 7. From 1990 to 2005, various land-use types within the study area exhibited varying degrees of interconversion. The direct conversions involved transitions from DRA, GRL, and UNL to NAW. Among all land-use types, NAW exhibited the most notable variation, characterized by a considerably larger area converted out compared to the area converted in. In total, 1.97 × 10^4^ km^2^ of natural wetlands was developed as DRA, PAF, and REP, with the most significant performance from the period 2000–2005. Meanwhile, 1.26 × 10^4^ km^2^ of NAW gradually transformed into WOL, GRL, and UNL. Additionally, a small proportion of NAW was developed from COL. On the basis of the land use transfer trajectory, it is evident that over the 15 years of continuous decline in NAW areas, there was a pronounced trend of strong conversion out. In contrast, other land cover types, including DRA, WOL, and GRL, exhibited a weaker trend of transformation out.

From 2005 to 2020, the proportion of land-use-type conversions increased, with NAW continuing to exhibit the most significant variation. However, unlike the period from 1990 to 2005, the proportion of NAW inflows to the total area exceeded that of outflows. The main land cover types that converted into NAW were GRL and WOL, with conversion areas of 2.01 × 10^4^ km^2^ and 4.24 × 10^4^ km^2^, respectively. Following these, DRL exhibited a notable trend, particularly from 2005 to 2010, with a conversion area reaching 1.57 × 10^4^ km^2^. Furthermore, a weak mutual conversion was observed between PAF, REP, COL, and NAW. During the 15 years of natural wetland area restoration, NAW showed a strong conversion in trend, while WOL and GRL showed a strong conversion out feature. Other land cover types showed varying degrees of turn-out phenomenon.

**Figure 8:**
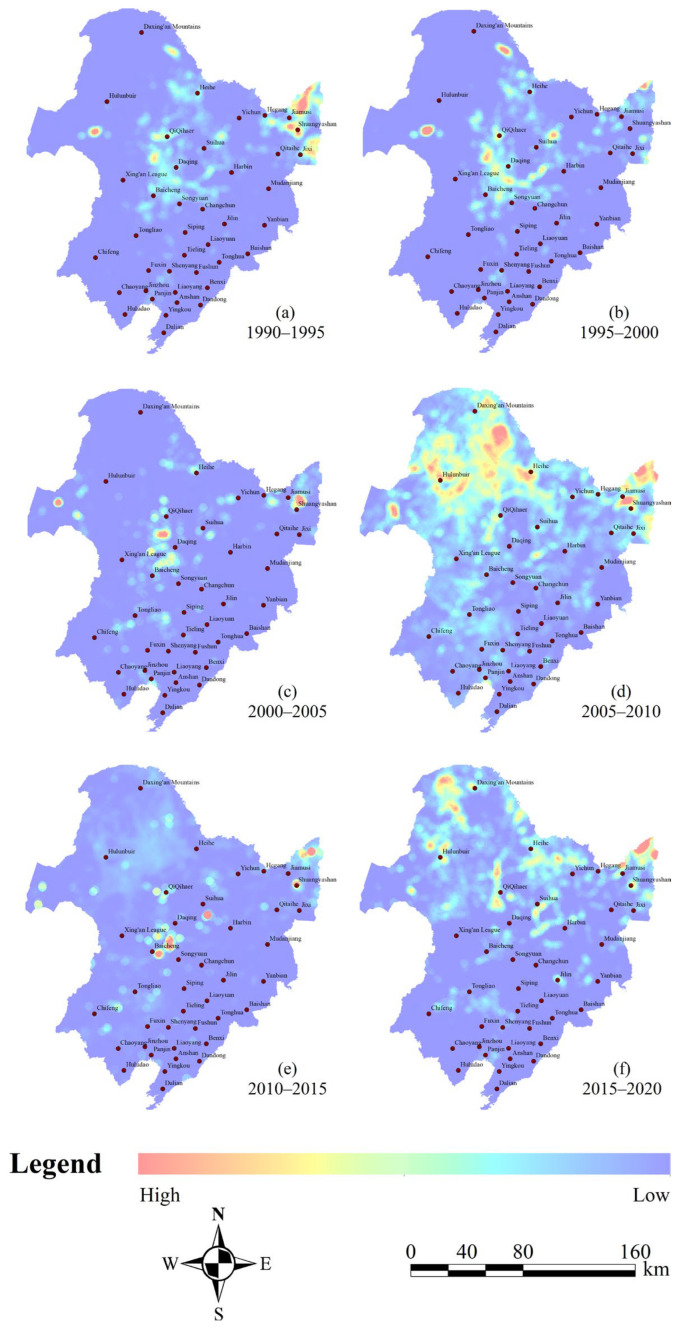
Distribution of spatiotemporal transfer intensity of different land types.

Although the transition matrix takes into account the internal mutual transformation areas between wetland types, it cannot directly reflect the changes in the spatial transformation intensity. Therefore, the grid overlay method was used to integrate the wetland variation data into specific spatial units for score calculation. According to the actual situation of the study area, the grid unit was set to 10 km × 10 km, and the dynamic change area of the wetland was overlaid on each grid unit through grid division. Then, the regional statistical method was adopted to count the wetland variation area in the unit grid and calculate the wetland transformation intensity. The spatial interpolation method was used to generate the spatial distribution map of the transformation intensity. Figure 8 indicates that the transfer intensity of natural wetlands during 2005–2010 and 2015–2020 was significantly higher in other periods. The areas with frequent conversions were concentrated in the northeastern part of the study area, specifically in the Yichun, Heihe, and Greater Khingan mountains. These regions exhibited higher transfer intensity, with the most notable changes observed in the Ussuri River Basin. In comparison, during 1990–1995 and 1995–2000, the changes in natural wetlands were mainly distributed in the central part of the study area, including Baicheng, Songyuan, and Qiqihar. Similarly, intense land cover conversions were also observed in the western part of the study area, specifically in the regions of Shuangyashan and Jiamusi. In contrast to other study periods, the regions with intensive land cover conversions during 2000–2005 and 2010–2015 often exhibited scattered patches. Although dramatic transitions were observed in the central and eastern parts of the study area, including Baicheng, Songyuan, and Shuangyashan, most areas undergoing drastic changes still displayed a pattern of central diffusion distribution, indicating a high spatial heterogeneity.

### 3.3. Quantitative Analysis of Natural Wetland Driving Forces

**Figure 9:**
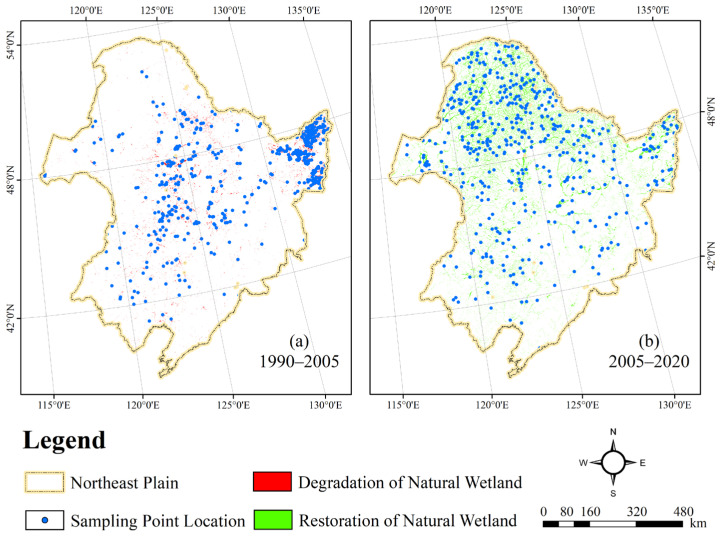
Spatial distribution of sampling points.

The study area was divided into 10 km × 10 km grid units using the grid unit method. The extracted natural wetlands variation density data and the data of the eleven driving factors were converted to the same scale and projected coordinate system. Rasterization was performed, and the spatial accuracy of the raster data was made consistent with the grid unit layer. Finally, a multisource information spatial database of natural wetlands variation in the study area was constructed. To avoid the influence of spatial autocorrelation on the accuracy of the training sample selection, this study adopted a spatial balance method from both temporal and spatial scales to calculate the spatial distribution weights of natural wetlands in the study area, effectively improving the representativeness of the sampling results. Based on the spatiotemporal evolution mechanism of natural wetlands in the Northeast Plain over 30 years, the study cycle was divided into degradation (1990–2005) and restoration (2005–2020). Two sets of 500 independent spatiotemporal sample points were obtained by setting weight thresholds and selecting sample points, each representing a distinct spatial distribution characteristic (Figure 9). Both datasets were constructed using 11 indicators, including ELE, SLO, TEM, PRE, DRN, PDD, GDP, DRA, PAF, WOL, and GRL as the input variables $x_{i}$ for the training samples, and the percentage of natural wetlands’ area variation was used as the output $y_{i}$ of the training sample to construct the training sample set $\left(x_{i},y_{i}\right)$.

**Figure 10:**
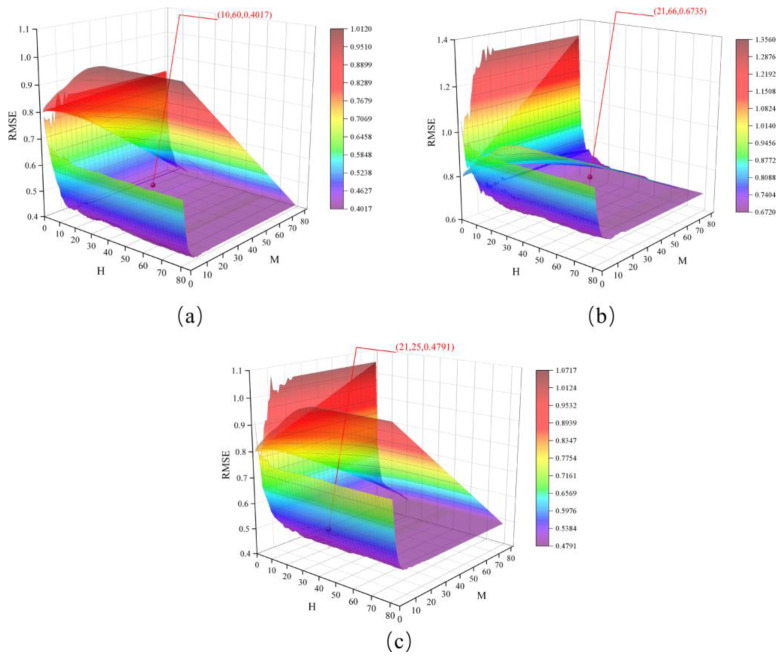
Parameter search results and training data distribution of the driving force analysis model: (**a**) parameter optimization of DEG-SSA-XGBoost; (**b**) parameter optimization of RES-SSA-XGBoost; (**c**) parameter optimization of COM-SSA-XGBoost.

According to the basic principles of the XGBoost model, the learning rate α (0 < α < 1), the iteration depth *H*, and the number of iterations *M* are three critical parameters that affect the performance of the XGBoost model. The learning rate α determines the possibility and period for the objective function to converge to a local minimum by adjusting the weights of the training iterations. The iteration depth *H* controls the training iterations, constrains the model fitting effect, and enhances the model’s generalization ability. The number of iterations *M*, also known as the number of decision trees, determines the number of split nodes involved in the iterative calculations. A larger value of *M* leads to more divided nodes participating in the iterations, potentially decreasing the model’s computational efficiency. In this study, the learning rate α was set to the default parameter of 0.3. Based on the given parameter space, the sparrow search algorithm (SSA) was utilized to search for the optimal values of the model’s critical parameters *H* and *M*. The root mean square error (RMSE) was set as the fitness value for the optimization objective function. The SSA algorithm possesses advantages such as a fast convergence speed, global solid search capability, and multiobjective optimization. Its initial parameters include the maximum number of iterations *G*, population size *P*, the ratio of discoverers to population size *PD*, number of scouts *SD*, and alarm value *R*_2_. In this study, the parameters for initializing the SSA algorithm were set as follows: *G* = 80, *P* = 50, *PD* representing 20% of the population size, *SD* = 5, and *R*_2_ = 0.8. This research chose these values as the default parameters for the SSA algorithm. The sample dataset was divided into training and validation sets in the experimental process according to an 8:2 ratio. The RMSE was used as the fitness function for the SSA algorithm. Combined with the XGBoost model, parameter training was conducted separately for natural wetlands’ degradation (DEG), restoration (RES), and complete (COM) cycle to obtain the optimal driving force analysis model. Figure 10 demonstrates a gradual decrease in the calculated RMSE values of the model as the iteration count increases. When *G* = 60, the DEG-SSA-XGBoost model achieves the lowest RMSE of 0.4017 during the natural wetland degradation period, with corresponding optimal values of iteration depth *M* = 10 and determination coefficient *R*^2^ = 0.759 (Figure 10a). When *G* = 66, the RES-SSA-XGBoost model attains the lowest RMSE of 0.6735 during the natural wetland restoration period, with corresponding optimal values of iteration depth *M* = 21 and determination coefficient *R*^2^ = 0.722 (Figure 10b). Lastly, when *G* = 25, the COM-SSA-XGBoost model exhibits the lowest RMSE of 0.4791 for the complete natural wetland cycle, with corresponding optimal values of iteration depth *M* = 21 and determination coefficient *R*^2^ = 0.697 (Figure 10c). 

**Figure 11:**
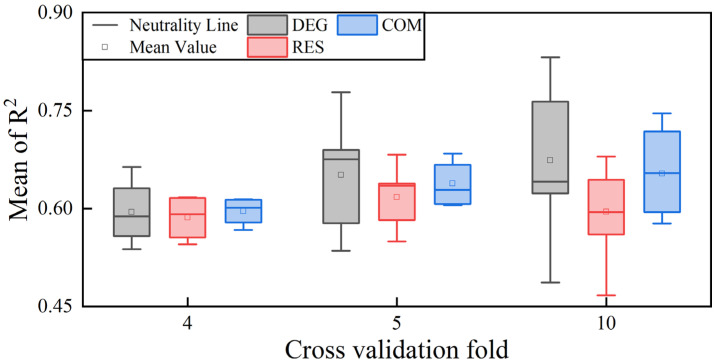
Multimodel accuracy evaluation results using a *k*-fold cross-validation.

To better evaluate the reliability of the driving force analysis model constructed by the optimal parameters, we employed the *K*-fold cross-validation method and used the coefficient of determination *R*^2^ as the evaluation metric for analysis. An analysis mechanism for *k* taking values of 3, 5 and 10 was constructed. From Figure 11, it can be observed that under different segmentation strategies, the average *R*^2^ of multiple models all exceeded 0.6, with the highest accuracy achieved under the fivefold cross-validation. At this point, the RES-SSA-XGBoost model exhibited a skewed phenomenon. Additionally, we found that compared to 4-fold and 5-fold, the 10-fold data distribution still had a median above 0.6, but it was more scattered, and the RES-SSA-XGBoost model showed the lowest lower limit. This indicates that the driving force analysis model constructed with the optimal parameters can handle outliers. However, in cases with a limited number of validation datasets, there may be a risk of local overfitting. The above results also indirectly confirm the rationality of conducting parameter optimization experiments with an 8:2 ratio.

Based on the training dataset, a driving force analysis model was constructed by optimizing the results using different parameters, which quantitatively evaluated the contributions of various driving factors to the degradation, restoration, and integrity periods of natural wetlands. According to the feature importance ranking of various driving factors by the XGBoost model (Table 2), it is evident that during the degradation period of NAW, agricultural activities represented by DRA and PAF had the highest contribution rates, reaching 30.55% and 22.44%, respectively. They were followed by topographic factors consisting of ELE and SLO, which had lower driving effects on natural wetlands, both of which were less than 10%. Finally, the contribution of meteorological factors represented by PRE and TEM was the lowest, with the contribution rate of PRE being the lowest at only 2.57%. It is worth noting that the DRN, as a primary indicator of human activities, exhibited a contribution rate comparable to that of geographical factors during the natural wetland degradation period, exceeding 8%.

For the natural wetland restoration period, both DRA and PAF were the main influencing factors for wetland variation. Among them, PAF showed a particularly significant impact with a contribution of 15.96%. Meantime, the contribution of GRL exceeded that of DRA and PAF, demonstrating a strong phenomenon of significant driving force transfer. Additionally, PRE exhibited a significant driving force during the natural wetland restoration period, with a contribution rate exceeding 10%. In the complete cycle of natural wetland variation, DRA and PAF became the main driving force of natural wetland dynamics. ELE and SLO profoundly influenced natural wetlands, while DRN and PRE were strongly associated with natural wetlands, respectively. GRL also played a driving role in natural wetland variation. GDP and WOL contributed the least to the evolution of natural wetlands.

## 4. Discussion

### 4.1. Spatiotemporal Variation of Natural Wetlands

**Figure 12:**
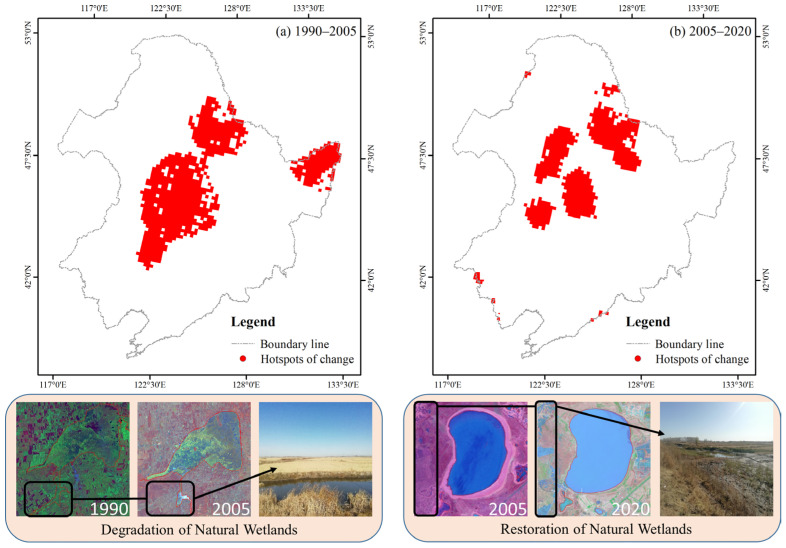
Hotspot areas of spatial and temporal changes in natural wetlands and fieldwork: (**a**) 1990–2005; (**b**) 2005–2020.

From 1990 to 2020, the natural wetland area in the Northeast Plain of China showed a decreasing trend followed by an increasing trend, with a turning point in 2005 (Figure 5). This result is consistent with the findings of Zhao et al. in 2018 [73]. As important driving factors in the spatiotemporal dynamics of natural wetlands, dryland and paddy fields have primarily concentrated in the central and northeastern regions of the study area over the past 30 years (Figure 2). In contrast to the relatively minor changes in the central region, the northeastern region represented by Shuangyashan and Jiamusi experienced frequent land-use conversion phenomena before 2005, making it a hotspot for changes (Figure 6 and Figure 12a). This includes the transformation mechanisms between natural wetlands and dryland, as well as natural wetlands and paddy fields, reflecting the high-density human intervention in that area (Figure 7 and Figure 12a). In the 1980s, China began to implement the household joint production responsibility system, which allowed farmers to lease arable land to families for 30 years and promoted the expansion of agriculture based on modern machinery. During the same period, residents in the central and eastern regions of the study area extensively expanded dryland and paddy fields at the expense of developing natural wetlands to increase income and alleviate the pressure of rapid urban–rural population growth. However, excessive agricultural reclamation led to abrupt changes in the living environment of some natural wetlands. The disappearance of much native flora and fauna in wetland ecosystems led to a decline in their ability to resist natural disasters, ultimately resulting in exacerbated soil erosion, frequent waterlogging and drought disasters, and other serious consequences. The promulgation of the “China Wetland Conservation Action Plan” in 2000 and the establishment of the first national wetland reserve in China, the “West Lake Wetland Reserve in Hangzhou, Zhejiang” in 2005, marked the beginning of the standardization of wetland conservation management system in China [74]. 

From 2005 to 2020, the most significant changes in natural wetlands in the Northeast Plain were mainly distributed in the central and western parts of the study area, which included Qiqihar, Xing’anmeng, and Harbin in the northern part (Figure 7 and Figure 12b). The land types that experienced significant dynamic changes were mainly woodland and grassland (Figure 6). During the study period, the overall decline rate of natural wetlands in the Northeast Plain was mitigated due to a substantial influx of forested land from the north and grassland from the east (Figure 2) [75]. This phenomenon may be related to the implementation of the “Five Small Projects” (small reservoirs, ponds, pumping stations, irrigation districts, and small wells) and the policy of returning farmland to forest/grassland by the local government. The “Five Small Projects” are supported by special funds for agricultural water conservancy, aiming to continuously build water conservancy facilities to expand the storage area of small- and medium-sized lakes and the water flow of main rivers (Figure 12b) [76]. On the other hand, the policy of returning farmland to forest/grassland is intended to achieve the self-repair of the natural wetland ecosystems by limiting human intervention in the natural environment and utilizing high-quality water sources [77]. Therefore, with the support of these policies, human agricultural activities in some areas have gradually reduced, and the natural wetland coverage has started to slowly rebound, as observed in the Hulunbeier and Daxinganling regions from 2005 to 2010.

### 4.2. Main Driving Force of Natural Wetland

**Figure 13:**
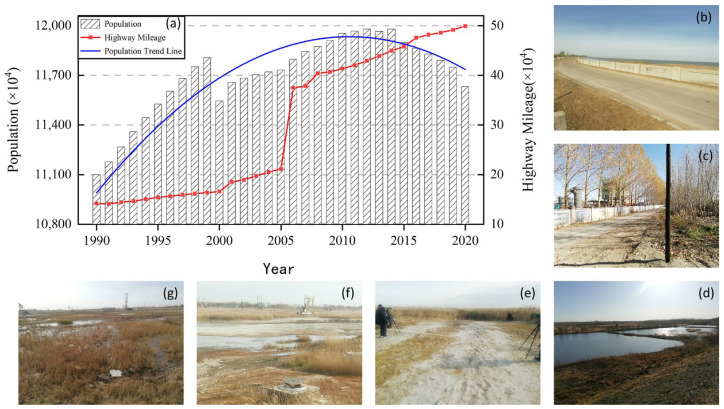
Dataset and field investigation photos documenting human drivers of wetland change: (**a**) Northeast China Plain’s highway mileage and population numbers; (**b**,**c**) roads and highways; (**d**,**e**) human development; (**f**) industrial activity; (**g**) degeneration of natural wetlands.

Human activities represented by dry land and paddy fields were the main factors causing variations in the area of natural wetlands in the research period. The contribution rate of the entire cycle reached 18.59% and 15.40%, and the dryland contribution rate in the decreasing period exceeded 30% (Table 2). This indicates that dry land is the most important factor involved in the spatial–temporal evolution process of wetlands and can be used to understand the fluctuation and changes of natural wetlands in the Northeast Plain. Usually, these changes are caused by human activities, especially agricultural activities in recent decades, such as population growth and migration, and an increase in infrastructure such as roads and bridges, etc. As shown in Figure 13a, the total population of the Northeast Plain during the research period showed an increasing trend followed by a decreasing trend, reaching a peak in 2012. Meanwhile, by analyzing Figure 4 and Figure 7, we can observe that the most significant spatiotemporal changes in natural wetlands during the study period occurred in the northeastern region of Jiamusi and the central region of Qiqihar, where the population is concentrated. This indicates a distribution trend with a high population density in the east gradually decreasing towards the west, and from south to north, which directly impacts the spatiotemporal dynamics of natural wetlands. It also demonstrates the substantial contribution of population density to the restoration period of wetlands. In contrast to population density, road network density, as another factor characterizing the impact of human activities on natural wetlands, has been concentrated in the central region of the Northeast Plain over the past 30 years. In some areas, it shows a spatial distribution pattern that is consistent with POP, GDP, and the spatial distribution trend of natural wetlands, all exhibiting a local aggregation phenomenon from south to north (Figure 4). This indicates a profound connection between the spatiotemporal dynamics of natural wetlands throughout the complete period and road network density. The low level of road construction mileage in China from 1900 to 2005 has also successfully demonstrated the significant impact of road network density on the high proportion of spatiotemporal dynamics during the wetland degradation period (Figure 13a). On the one hand, the surge in population has brought about a huge demand for food, and the inefficient road network cannot achieve the inter-regional circulation of limited social resources. This has compelled people to initiate large-scale road network construction, resulting in the encroachment of natural wetland coverage (Figure 13b,c) [78]. It is worth noting that the expansion of the road network can, in reverse, drive the migration of population spatial agglomeration centers, leading to the extensive conversion of local areas with abundant natural wetlands into arable land, thus alleviating the growing pressure on food production (Figure 12a). On the other hand, the establishment of road networks and population growth, while promoting rapid regional economic development, also brings about serious water consumption issues (Figure 13d). By analyzing Figure 4 and Figure 7, it can be inferred that the high GDP in the central and eastern regions of the study area is likely achieved at the expense of sacrificing natural wetlands. This impact mechanism is often indirect, mainly manifested in the significant increase in agriculture, industry, and human water consumption (Figure 13e,f). Excessive demand for water resources can lead to a rapid decline in the groundwater content of the natural wetland cover area, and the vertical shallow cycle system of groundwater supply–runoff–discharge has been seriously affected, resulting in a weakened atmospheric circulation and drastic climate changes in the area [79,80]. Eventually, some natural wetlands form near lakes and rivers shrink, which triggers a decline in the area covered by natural wetlands (Figure 13g).

**Figure 14:**
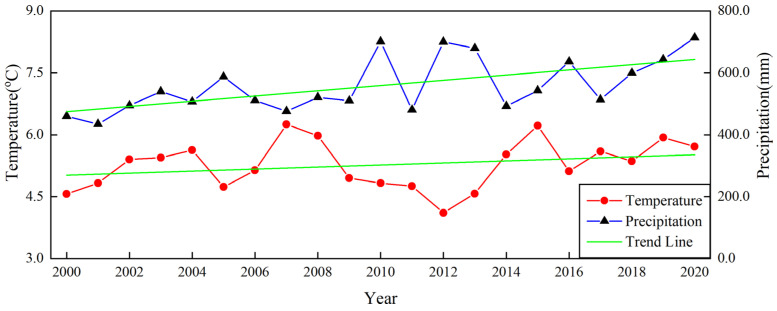
Trends of climate factors in the Northeast China Plain from 2000 to 2020.

In addition, natural factors consisting of meteorological and topographic factors also have a certain driving effect on natural wetlands. In terms of meteorological factors, the increasing trend of precipitation and temperature from the northwest to the southeast in the study area reflects a significant positive correlation between the spatiotemporal dynamics of natural wetlands and meteorological factors at the spatial scale. Meanwhile, the upward trend in annual precipitation and temperature in the Northeast Plain over the past 20 years shown in Figure 14 also reveals the existence of strong precipitation cycles associated with the greenhouse effect at the temporal scale. This ultimately confirms the spatiotemporal relationship between meteorological factors and the spatial distribution of natural wetlands during the wetland recovery period. During this period, the wetland area in the Northeast Plain of China increased by 37.76 × 10^3^ km^2^, possibly due to the increased surface runoff replenishment caused by abundant precipitation and balanced temperature, and the reasonable transpiration promoted the energy cycle of the natural wetland aquatic ecosystem, triggering a new round of natural wetland self-ecological restoration [81,82,83]. From the perspective of topographic factors, the uneven distribution of elevation can lead to changes in surface water runoff direction, thereby affecting the spatial distribution of natural wetland resources. As shown in Figure 4, the study area is characterized by an east-high–west-low and north-high–south-low topographic pattern, with predominantly low-altitude plains having a slope lower than 0.5, widely distributed in the central and eastern regions of the Northeast Plain. In addition, the natural wetlands are generally located on a flat, low-altitude plain of the study area. The climate in that area is suitable, and the water resources are abundant, which can meet the needs of wetland plant communities (Figure 4 and Figure 7). It especially has favorable topographic conditions for the survival of aquatic plants. Therefore, this may be the main reason why topographic factors drive the spatiotemporal evolution of natural wetlands [84,85].

### 4.3. Driving Force Analysis

The rational selection of driving factors is the key to building a driving force analysis model. The natural wetland ecosystem is a giant system in which natural, economic, social, and other factors differ spatially. Therefore, the driving mechanism of its spatiotemporal evolution trend must consider the influence of spatial heterogeneity. In this paper, we borrowed from the research results of Zhang et al. and selected traditional numerical data such as dry land, paddy fields, and population density as the basis for driving factors [55]. We also introduced road network density to enrich the overall structure of the influencing factors and used spatial analysis methods to perform a diversified processing of raster data, achieving the joint characterization of time and space dimensions based on a spatial balance sampling method. The road network density is composed of multiple levels of highways and railways. Its large-scale construction generally involves filling wetlands, excavating rivers, and digging channels. This will seriously change the water source quality and hydrodynamic characteristics of the nearby natural wetland ecosystem, leading to soil erosion and restricted vegetation growth in cultivated land. Therefore, using road network density as a driving factor for the spatiotemporal evolution of natural wetlands and participating in building a driving force analysis model has a strong theoretical basis.

In this paper, we used the SSA and XGBoost algorithms with RMSE as the fitness function to obtain the optimal driving force model through multiple rounds of iterative training. The results showed that for both the decreasing and rising periods of natural wetlands, the SSA algorithm could quickly locate the internal key parameters of the XGBoost algorithm. The optimal parameter construction model verification set’s *R*^2^ exceeded 0.7, which could achieve the expected goal of quantifying the contribution rate of driving factors and obtaining the main driving forces of the spatiotemporal evolution of natural wetlands in the Northeast Plain. Furthermore, the conducted k-fold cross-validation experiments also confirmed the effectiveness of the SSA algorithm’s parameter results and revealed that inappropriate dataset splitting ratios could perturb the model performance (Figure 10). It is worth noting that the XGBoost algorithm, as a classical shallow machine learning algorithm, mainly uses the decision tree model as a weak learner, integrates and calculates the optimal path corresponding to each leaf node, and extracts feature weights (contribution rates) to obtain the main driving forces. Its analysis results not only agree with the traditional driving force models of Wang et al. and Ge et al. using a linear regression but also effectively avoid the overfitting problem caused by the endogenous collinearity of driving factors through regularization techniques. This confirms the universality and feasibility of machine learning theory in analyzing the spatiotemporal evolution driving forces of natural wetlands.

## 5. Conclusions

In this paper, we used the dynamic index, transition matrix, and transition intensity methods to explore the spatiotemporal evolution trend of natural wetlands from 1990 to 2020 in the Northeast Plain. We selected meteorological factors, topographic factors, socio-economic factors, and typical land-use types, embedded space factors based on the spatial balance sampling method, and constructed an SSA-XGboost hybrid driving force analysis model coupling time and space dimensions. A quantitative analysis of the correlation between each driving factor and the changes in natural wetlands was performed. The main research results are as follows:

(1) In the past 30 years, the spatiotemporal evolution of different land-use types in the Northeast Plain has been relatively drastic, forming a multilevel land-use structure change mainly composed of dry land, forest land, grassland, and natural wetland. The overall area of natural wetlands has shown an upward trend, with an increase of 32%, and a sudden change occurred between 2005 and 2010, with a leap amplitude close to 50%. At that time, frequent transformations between land-use types occurred.

(2) During the research period, the transfer characteristics among different land-use types in the Northeast Plain were evident. From 1990 to 2005, the natural wetlands exhibited a strong transfer-out feature, especially from 2000 to 2005, with a development area of 1.97 × 10^4^ km^2^, mainly concentrated in the central part of the study area, including Baicheng, Songyuan, and the eastern part of Shuangyashan. From 2005 to 2020, the grassland and forest land were the main sources of transfer-in for natural wetlands. There was also a phenomenon of dry land transfer-in of 1.57 × 10^4^ km^2^ from 2005 to 2010, mainly located in the northeastern part of the study area, including Yichun, Heihe, and the Greater Khingan Range.

(3) Based on the spatial balance sampling method, incorporating spatial factors into time series data can effectively achieve the joint characterization of driving factor data in time and space dimensions. By deeply integrating the SSA algorithm with the XGBoost model, we effectively avoided the issue of multicollinearity among the driving factors and achieved a rapid parameter estimation for the model. From the perspective of relative contribution rate, the dry land and paddy fields were the main influencing factors throughout the whole period, while elevation, precipitation, and road network density became secondary factors for the changes in natural wetlands. Other driving factors increased the complexity and heterogeneity of wetland change driving force analysis.

## Figures and Tables

**Table 1:** Seven phase data on land area of Northeast Plain (×10^3^). Unit: km^2^, %.

Year	DRA	PAF	WOL	GRL	UNL	COL	NAW	REP
1990	284.32	34.10	519.40	259.02	79.52	26.20	79.27	3.38
1995	317.91	38.81	504.09	240.28	70.01	29.73	72.13	3.37
2000	323.84	45.00	499.36	234.54	69.88	29.15	68.72	3.81
2005	325.65	44.90	499.26	234.05	68.98	29.57	67.03	3.83
2010	319.48	52.54	504.56	188.01	104.74	33.87	98.78	4.02
2015	317.36	55.48	504.43	186.64	104.41	34.57	98.40	4.06
2020	321.01	64.95	498.93	169.99	113.37	37.46	104.79	5.30

**Table 2:** Results of driving force analysis for the SSA-XGboost model. Unit: %.

Driving Factor	1990–2005 (10, 60, 0.4017)	2005–2020 (21, 66, 0.6735)	1990–2020 (21, 25, 0.4791)
ELE	9.77	6.82	10.59
SLO	9.93	6.95	10.88
PRE	2.57	10.05	8.62
TEM	4.1	6.8	5.90
POP	2.6	8.38	5.98
GDP	3.31	4.14	2.45
DRN	8.38	5.97	9.35
DRA	30.55	15.04	18.59
PAF	22.44	15.96	15.40
WOA	1.79	3.08	1.87
GRL	4.56	16.81	10.37

## Data Availability

Restrictions apply to the availability of these data. Data was obtained from [Chinese Academy of Sciences] and are available [http://www.resdc.cn/DOI, accessed on 1 July 2021] with the permission of [Chinese Academy of Sciences].
